# Utility of Baseline Pathological, Neuroimaging and Clinical Markers for Prognosis in Early Parkinson’s Disease

**DOI:** 10.1177/08919887251397641

**Published:** 2025-11-13

**Authors:** Angus McNamara, Benjamin Paul Ellul, Irina Baetu, Mark Jenkinson, Stephan Laurenz, Lyndsey Collins-Praino

**Affiliations:** 1 School of Biomedicine, 1066 University of Adelaide, Adelaide, SA, Australia; 2 School of Psychology, 1066 University of Adelaide, Adelaide, SA, Australia; 3 Australian Institute for Machine Learning, School of Computer and Mathematical Sciences, 1066 University of Adelaide, Adelaide, SA, Australia; 4 South Australian Health and Medical Research Institute, Adelaide, SA, Australia

**Keywords:** Parkinson’s disease, biomarkers, neuroimaging, subtyping, prognosis

## Abstract

**Background:**

Currently, prognosis of Parkinson’s Disease (PD) is limited. Emerging literature highlights potential of multi-modal biomarkers and neuroimaging to provide critical insight into clinical progression, potentially improving prediction of long-term outcomes.

**Methods:**

Data were extracted from the Parkinson’s Progression Markers Initiative (PPMI). Hierarchical clustering was applied to Movement Disorder Society Unified Parkinson’s Disease Rating Scale (MDS-UPDRS) scores at year-five follow-up, identifying two clusters. Differences in progression, as well as retrospective assessment of baseline differences, between clusters were explored for pathological biomarkers, neuroimaging, and prodromal measures. Additionally, logistic regression, receiver operating characteristic curve analyses and machine learning were employed to determine utility of variables at baseline as predictors of cluster membership.

**Results:**

The more impaired cluster demonstrated worse motor and non-motor outcomes, including higher rates of dementia and cognitive complaints at year-five, as well as more profound rigidity than cluster one. Further, retrospective comparisons showed cluster two performing worse in all prodromal measures and demonstrated lower striatal dopamine transporter and cognitive ability. Logistic regression determined that membership in this cluster was predicted by higher autonomic dysfunction and p-tau, along with reduced smell and alpha-syn, predicting 49.1% of variance (AUC = 0.92). This was significantly higher (*p* < 0.001) than the model including MDS-UPDRS scores alone, only accounting for 27.4% of variance (AUC = 0.74). Findings were corroborated by machine learning, whereby multi-modal assessment corresponded to 74% classification accuracy, compared to 60% with MDS-UPDRS alone.

**Conclusion:**

Prediction of more marked impairment at year-five was substantially improved via multi-modal assessment, specifically, pathological biomarkers, suggesting that incorporating biomarkers into clinical criteria could enhance long-term prognosis.

## Background

The official Movement Disorder Society (MDS) Clinical Diagnostic Criteria for Parkinson’s disease (MDS-PD criteria) were proposed in 2015,^1^ presenting high sensitivity and specificity.^2^ Although these criteria incorporate non-motor symptoms, motor symptoms remain the core feature of MDS-PD diagnosis, specifically bradykinesia and either resting tremor or rigidity.^1^ A widely implemented clinical rating scale for assessing PD features, including motor impairment, is the Movement Disorders Society Unified Parkinson Disease Rating Scale (MDS-UPDRS).^3^ The MDS-UPDRS is also widely utilised to identify subtypes of PD, a critical consideration for personalised treatment and management, as PD subtypes display differential outcomes and rates of progression (see review,^4^). Despite this, however, recent research strides highlight the need to extend beyond conventional clinical assessment and employ a more comprehensive strategy to better encompass the complex nature of PD and potentially improve diagnosis and the forecasting of long-term outcomes and progression.^5^

To mitigate such challenges, the incorporation of biomarkers, as is currently employed for Alzheimer’s Disease (AD), may offer a path forward.^6^ Like PD, AD is a neurodegenerative disorder, with diagnosis originally focused on clinical symptoms, namely cognitive impairment.^7,8^ Later research established AD pathology present in the brain several years prior to clinical onset.^9^ Accordingly, the A/T/N framework (“A” - β-amyloid (Aβ); “T” – phospho or total tau and “N” – biomarkers of neurodegeneration and neuronal injury) was developed as a biomarker-focused framework assessing pathological hallmarks of AD and their utility for diagnosis.^6,10^ Preliminary assessment of these criteria indicate higher discriminative accuracy of AD vs cognitively unimpaired adults and other neurodegenerative diseases,^11^ along with high utility for predicting cognitive decline.^12^

Success seen with the A/T/N framework suggests incorporating multi-modal markers into PD criterion may be similarly beneficial. Currently, however, PD diagnostic criteria is based almost entirely on clinical judgment, with cardiac sympathetic denervation on meta-iodo-benzyl-guanidine (MIBG) scintigraphy being the only laboratory test included as a diagnostic criterion.^1^ However, recent developments, such as the alpha-synuclein (α-syn) seed amplification assay (SAA), proposed to have utility in differentiating those with PD from controls (see review,^13^), challenge this notion. In support of this, a cross-sectional study of 1123 participants in the Parkinson’s Progression Markers Initiative (PPMI) cohort by Siderowf and colleagues^14^ (2023) demonstrated that α-syn SAA could classify PD with high sensitivity and specificity. Importantly, α-syn SAA could detect prodromal individuals prior to diagnosis,^14^ with 86% of individuals with REM sleep behaviour disorder or olfactory impairment, 2 common prodromal symptoms, showing a positive α-syn SAA. Similarly, it has been proposed that neuronally-derived extracellular vesicle α-syn (ie, L1EV-associated α-syn) may be a potential biomarker for PD.^15^ In a recent cross-sectional study of 576 individuals, Yan and colleagues reported that L1EV α-syn differentiated high-risk participants (>80% probability of developing PD) from those with low-risk or matched controls. Further, in a cohort of 40 who later developed PD and related dementia, blood tests were positive for L1EV α-syn in more than 80%,^15^ further supporting the use of biomarkers to advance PD diagnosis and prognosis. Such findings are reflected in the recent development of the Neuronal α-syn Disease (NSD) framework, exploring underlying biology as a staging system of PD.^16^

In addition to biological fluid-based biomarkers, neuroimaging may also help to improve diagnosis and prognosis in PD. While a positron emission tomography (PET) tracer for detecting α-syn pathology is in development (^18^F ACI-12589), it is not yet validated for clinical use.^17^ However, other PET tracers have shown promise, such as Flourodopa F18 (F-Dopa), recently approved by the Food and Drug Administration to aid in earlier diagnosis of PD.^18^ Encouragingly, F-Dopa imaging yields high correlations with PD motor outcomes and severity.^19^ Additionally, conventional T2-weighted MRI has been used to detect degeneration of regions known to be affected during PD. For example, early clinical studies have reported significantly higher substantia nigra *pars compacta* (SNc) iron content in PD compared to healthy controls.^20,21^ Such elevations in iron content are proportional to disease severity, highlighting the potential prognostic utility of MRI.^21^

Given these findings, this study aimed to determine whether a comprehensive multi-modal approach, incorporating neuroimaging and biofluid markers, possessed utility in improving prognosis in PD beyond baseline MDS-UPDRS assessment alone. This study utilised data from PPMI to conduct data-driven subtyping on MDS-UPDRS scores 5-year after baseline assessment in an early PD cohort. The predictive utility of neuropathological measures of PD, including biological-fluid and neuroimaging-based biomarkers, in conjunction with prodromal assessments and current clinical criteria, to predict cluster membership was explored.

## Methods

### Participants & Materials

Analysis used data openly available from the Parkinson’s Progression Markers Initiative (PPMI),^22^ with the cohort of interest consisting of recently diagnosed PD participants (n = 422). A description of PPMI participant selection criteria can be found in Table S1. An overview of extracted data can be found in Table 1. Only the first 5-year’s worth of follow-up data were included in this study, in order to minimise risk of attrition bias.

**Table 1.** Data Extracted From the PPMI Database and Their Corresponding Assessments.

Category	Measures
Baseline demographics	Age
	Sex
	Education
Imaging modalities	T2-weighted MRI
	Obtained via a siemens 3T TIM trio scanner model at baseline
	Manual masking protocol (further information below) was conducted on scans to delineate regions of interest, acting as a proxy marker of SN volume
	DaT SPECT
	Participants were injected with radionuclide ligand DaTScan at baseline. Specific binding ratio of mean striatum (average of left and right caudate + putamen) was the measure of interest for this analysis
Biofluid pathological markers	Cerebrospinal fluid (CSF) (pg/mL)
	Obtained via standard lumbar puncture and processing protocols at baseline
	Measured using standard ELISA protocols
	- Unphosphorylated alpha synuclein (*α*-syn)
	Measured using elecsys electrochemiluminescence (ECL) immunoassays
	- Phosphorylated tau (phosphorylation site: threonine 181) (p-tau)
	- β-amyloid^1-42^ (Aβ)
	**S**erum (ng/mL)
	Obtained via standard venepuncture and processing protocols at baseline
	Measured using standard ELISA protocols
	- Insulin growth factor (IGF-1)
Autonomic function	Scales for outcomes in PD-autonomic (SCOPA-AUT)
	The SCOPA-AUT is a self-reported questionnaire with 25 questions, used to assess autonomic function in the following domains^23^
	1) Gastrointestinal
	2) Urinary
	3) Cardiovascular
	4) Thermoregulation
	5) Pupillomotor
	6) Sexual
	Collected at baseline
Sleep	REM sleep behaviour disorder questionnaire (RSBDQ)
	A 10-item, self-report questionnaire covering the clinical features of REM sleep behaviour disorder (RBD)^24^
	Collected at baseline
Smell	University of Pennsylvania smell identification test (UPSIT)
	An odour recognition test with 40-items, it is routinely used across a variety of olfactory disorders on account of its sensitivity and replicability^25^
	Collected at baseline
Motor symptoms & disease severity	Movement disorder society-unified Parkinson’s rating scale (MDS-UPDRS)
	A comprehensive questionnaire containing 50 items pertaining to motor and non-motor symptoms of Parkinson’s and is a combination of clinical assessment and self-report.^26^ This was extracted at baseline and at annual follow-ups up to 5 years while participants were on medication. The following components were used
	Part 1: Non-motor experiences of daily living
	- Total score
	Part 2: Motor experiences of daily living
	- Total score
	Part 3: Motor examination
	- Rigidity score (sum of MDS-UPDRS subitems of item 3.3)
	- Tremor score (sum of MDS-UPDRS subitems of items 3.15, 3.16, 3.17 & 3.18)
	Hoehn and Yahr staging scores at year-five was also extracted
Cognitive ability	Various aspects of cognition were assessed at baseline at annual follow-ups up to 5 years, as changes in cognition are widely recognised within PD.
	Hopkins verbal learning test (HVLT)
	- Verbal recognition and recall (learning and memory)
	Semantic fluency (SFT)
	- Executive function and semantic memory
	Letter number sequencing (LNS)
	- Working memory
	Benton judgement of line orientation (BJLOT)
	- Visuospatial ability
	Symbol digit modalities (SDM)
	- Psychomotor/Processing speed
	Cognitive assessment, along with PPMI investigator rating and information from external sources (family or friends), also acted as the basis for determination of cognitive disorders (ie, subjective complaint, PD-MCI, PD-D). Categorisation was extracted at year-five follow-up
Mood dysfunction	Various aspects of mood were assessed at baseline at annual follow-ups up to 5 years, as changes in mood are widely recognised within PD.
	State-trait Anxiety inventory (STAI)
	- Anxiety
	Geriatric depression score (GDS-15)
	- Depression

### Hierarchical Clustering

Improved characterisation of PD outcomes is critically needed, with a proposed strategy being data-driven subtyping.^27^ Showing promise is hierarchical clustering, which displays several advantages over other methods, such as K-means, (see review,^28^). Therefore, this study utilised enhanced agglomerative hierarchical clustering using Euclidean distance, conducted on year-five MDS-UPDRS scores, a non-biased approach to identify PD subtypes. Per recommendations to incorporate both motor and non-motor features,^29^ clustering inputs included total scores from MDS-UPDRS parts 1 and 2, to capture self-repot of both non-motor and motor experiences of daily living, respectively, as well as clinically-rated motor features, specifically rigidity and tremor scores derived from part 3 of the MDS-UPDRS, at year-five follow-up. Details on how scores were derived are found in Table 1.

While other motor symptoms, namely gait and postural stability, were considered for inclusion, a recent review by Bloem and colleagues reported that clinical rating scales such as the MDS-UPDRS perform sub-optimally when assessing such features.^30^ Additionally, MDS-UPDRS derived postural instability – gait disturbance (PIGD) scores lack sensitivity to change, and may not be suitable for mild PD, of particular relevance given this study assessed *de novo* participants.^30^ Therefore, gait and postural stability were omitted to minimise biasing clustering outputs. A recent study demonstrated that a score based solely on tremor and rigidity features was sufficient to identify a clinically relevant subtype.^31^ Additionally, the same rigidity score has already been used within the PPMI dataset, demonstrating sensitivity to change over time,^32^ supporting its use within the current study.

The decision to include only variables from the MDS-UPDRS in the clustering analysis was made a priori, given that this scale is the gold standard for assessing motor symptoms in a research context.^33^ While it was acknowledged that performance on measures of aspects of non-motor function, such as cognition and mood, might also be relevant for clustering at year 5 follow-up, these are not explicitly included within the MDS-UPDRS (although part 1 of this scale assesses self-report of non-motor aspects of experiences of daily living) and composite scores derived from these measures have not been clinically validated. Notably, however, our group has recently reported on baseline predictors of disease progression, based on fuzzy C-means clustering of year-5 cognitive and affective function scores, within the PPMI dataset.^34^

Clustering was performed using the *hclust* function within *factoextra*,^35^ and a phylogenetic tree was generated using the *fviz_dend* function. Prior to clustering, MDS-UPDRS data were converted into z-scores, and the number of clusters was determined via the *NbClust* package,^36^ with the optimal number of clusters identified in this case being 2, illustrated in dendrogram format (Figure S2).

### Principal Component Analysis

Principal component analysis (PCA) was conducted on cognitive assessments to develop a single composite score of cognitive ability, and separately on STAI and GDS as a measure of mood dysfunction. This was used to develop baseline cognition and mood scores, as well as a composite score reflecting the change in cognitive ability and mood dysfunction over time, by conducting PCA on the slopes of each neuropsychiatric assessment. Loadings confirmed that assessments were highly correlated, making it appropriate to convert into a single factor (Table S3).

### MRI ROI Delineation

Axial T2-weighted MRI scans were used to assess the structural quality of regions in proximity to the SN. Scans (n = 135) were acquired using a Siemens 3T TIM trio scanner, with a 12-channel matrix head coil and total acquisition time of 5 minutes and 8 seconds. Phase encoding direction was L/R (TE = 11 ms; TR = 3000 ms), with an acquired matrix size of 228 × 256 × 48 and voxel dimensions being 0.93 × 0.93 × 3 mm^3^.

**Figure 1.**
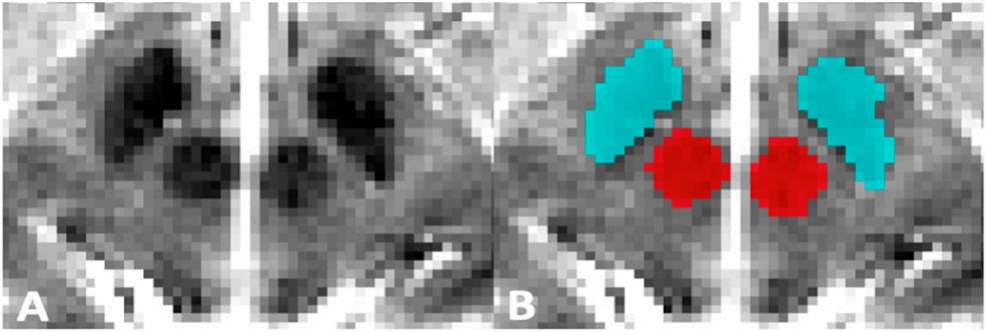
(A) Cropped midbrain region in an axial T2-weighted MRI, along with manual masks (B) for the SN-related T2 hypointensity (blue) and RN (red).

The SN was evaluated using a 2D proxy marker, the hypointense region comprised of crural fibres and anterosuperior portions of the SN.^37^ Accordingly, we have adopted the term “SN-related T2 hypointensity” to describe this marker. The red nucleus (RN) was chosen as a control region, due to possessing A10 dopaminergic neurons, which, despite being adjacent to the SN, are spared during PD.^38^ FSLeyes, an image viewing programme available in the FSL package,^39^ was used to create manual delineations of the ROI. Spatial masks were obtained bilaterally from 2 independent raters (Figure 1) and were averaged. SN-related T2 hypointensity and RN volume values were converted into ratios, which then accounts for the global effect of individual head size. Larger ratios corresponded to a larger SN-related T2 hypointensity and, consequently, more advanced disease.

### Statistical Analyses

All data were analysed via R (version 1.4.1717).^40^ A Shapiro-Wilk test determined several variables deviated significantly from normality, with only striatal DaT binding the SN-related T2 hypointensity being normally distributed. Normally distributed variables are presented as mean ± standard deviation, whereas non-normally distributed variables are reported as median (interquartile range), along with categorical outcomes displayed as counts (percentage).

As cluster membership was determined via MDS-UPDRS scores at year-five follow-up, retrospective assessment was conducted to determine whether differences between clusters were present at baseline using Mann-Whitney U tests, or T-tests for normally distributed data. To account for the effect of attrition, group differences for all baseline variables were assessed between participants included in the cluster analysis and those lost to follow-up. Further, differences in assessment scores at year-five, as well as clinical features such as Hoehn and Yahr staging and clinician determined cognitive outcomes, were compared between clusters. If data included baseline and corresponding follow-up, a non-parametric equivalent of a mixed ANOVA was conducted via the *nparLD* package,^41^ with a between-subjects factor (cluster membership) and a within-subjects factor (year). If a significant interaction effect was present, differences between clusters in motor, mood, and cognition slopes (indicating the extent of degeneration in both motor and non-motor outcomes over the follow-up period) were assessed via Mann-Whitney U tests.

Logistic regression was conducted to determine if baseline variables of interest predicted cluster membership, displayed using the *sjPLot* package.^42^ Two models were compared: 1) baseline MDS-UPDRS scores (the same measures whose year-five values were used as inputs for the clustering algorithms) and 2) baseline MDS-UPDRS scores, as well as demographic data, biomarkers and prodromal autonomic outcomes measured at baseline. An ANOVA was run between models to determine whether the inclusion of these additional variables significantly improved predictive accuracy beyond MDS-UPDRS alone. Further, receiver operating characteristic curve analyses were conducted for each model to determine area under the curve (AUC) values as measures of model performance. Both models only included participants with complete data (n = 111). The relative importance of each predictor was calculated via the *Caret* package.^43^ Importantly, regression models did not include the PCA-derived cognitive ability and mood dysfunction scores, as these are not clinically validated scores and are not derived from measures included within the MDS-UPDRS. Instead, these composite scores were utilised as external validation measures to corroborate the clustering outputs.

### Machine Learning

**Figure 2.**
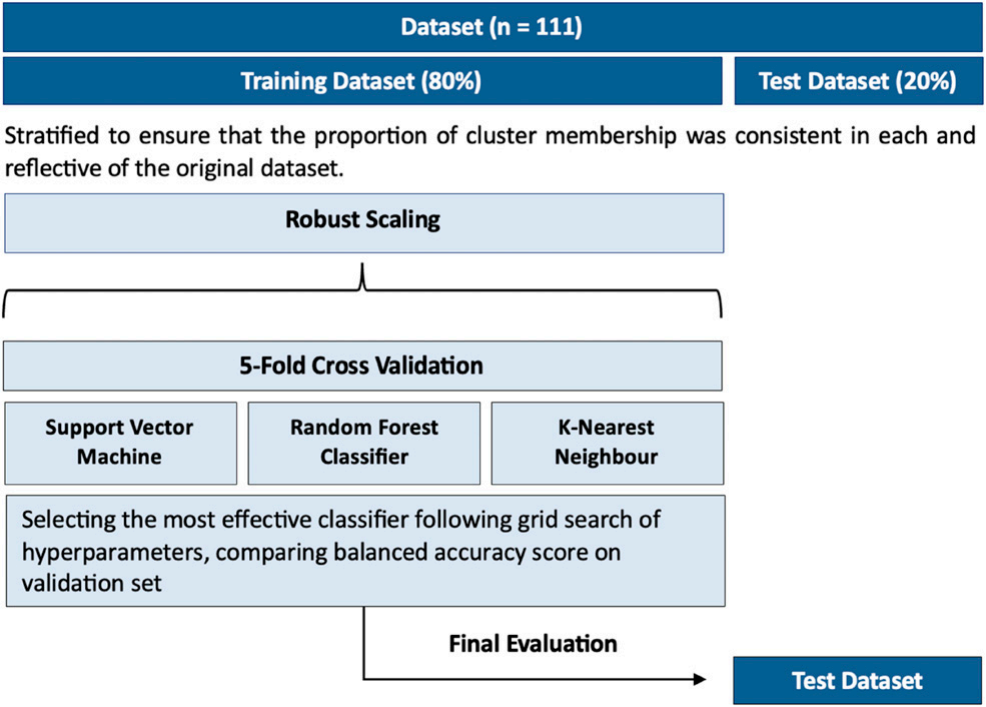
A 5-fold cross-validation pipeline was developed to create models using identical predictor inputs as used to develop the logistic regression models.

A stratified cross-validation pipeline was developed in Python (Figure 2) using the *scikit-learn* package.^44^ Models with identical predictor inputs to the logistic regression models were developed, incorporating 3 classifiers: support vector machines (SVM) (kernel = radial basis function), k-nearest neighbours (neighbours = 40, range = 2-60), and random forests (estimators = 100, range = 2-60) Balanced accuracy scores were compared to chance performance (50%), and between each classifier to determine the best classifier per predictor input to use on the test dataset. The final test result was compared to chance performance to determine the degree that the chosen machine learning classifier improved predictiveness. Overall accuracy in predicting cluster membership was compared to the corresponding logistic regression models, to determine whether the non-linearity feature of the machine learning models improved classification confidence.

## Results

### Baseline Comparisons

Cluster 2 demonstrates a significantly higher *(p =* 0.02) age (median = 63.5) compared to cluster 1 (median = 61.2), with both clusters reporting a higher proportion of males than females (cluster 1 = 63%; cluster 2 = 76%). In brief, cluster 2 displayed significantly worse performance in multiple MDS-UPDRS domains, including higher part 1 and 2 scores, as well as higher mood dysfunction (Table 2). Further, cluster 2 performed significantly worse in all prodromal assessments, along with significantly Lower striatal DaT binding and cognitive ability.

**Table 2.** Differences Between Clusters in all Extracted Assessments were assessed Retrospectively at Baseline. Normally Distributed Variables are Displayed as mean ± SD, With Statistical Differences Determined via T-Tests. Non-Parametric Variables are Displayed as Median (IQR), With Statistical Differences Determined via Mann-Whitney U tests. Count Data are Presented as Percentages (%) and Differences were Assessed via a Chi-Square Test. A Bonferroni Correction was Applied to Account for Multiple Comparisons, Setting the Threshold to P < 0.003. Statistically Significant Differences are Denoted by Bolded *P*-Values.

	Cluster 1 n = 221		Cluster 2 n = 79		*P*-value
	n	Mean ± SD/Median (IQR)	n	Mean ± SD/Median (IQR)	
Age	221	61.2 (54.7 – 67.9)	79	63.5 (66.9 – 70.9)	0.02
Education	221	16 (14 – 18)	79	16 (14 – 18)	0.39
Sex					
Female	81 (37%)		19 (24%)		0.06
Male	140 (63%)		60 (76%)		
MDS-UPDRS part 1	221	4 (2 – 6)	79	7 (5 – 10)	**< 0.0001**
MDS-UPDRS part 2	221	4 (2 – 6)	79	9 (5 – 12.75)	**< 0.0001**
Rigidity score	221	3 (2 – 5)	79	4 (2.25 – 6)	0.03
Tremor score	221	4 (2 – 6)	79	3 (2 – 5)	0.09
Cognitive ability	220	0.27 (−0.42 – 0.88)	79	−0.03 (−0.81 – 0.37)	**0.001**
Mood dysfunction	221	−0.35 (−0.77 – 0.27)	79	0.16 (−0.58 – 0.84)	0.004
CSF α-syn	216	1375.5 (1062.9 – 1854.2)	79	1322.6 (1036 – 1794)	0.17
CSF p-tau	197	13.19 (11 – 17.1)	71	13.48 (11.8 – 17.1)	0.46
CSF Aβ	214	848.6 (650.2 – 1195.8)	78	906 (671.30 – 1157.3)	0.73
Serum IGF-1	215	137.70 (103.3 – 166.2)	76	132.2 (99.3 – 176)	0.78
UPSIT	221	23 (17 – 29)	79	19 (12 – 26)	**0.002**
RBDSQ	221	3 (2 – 5)	79	5 (2 – 7)	**0.0008**
SCOPA-AUT	218	7 (4 – 10)	78	13 (10 – 17)	**< 0.0001**
SN proxy ratio	101	1.27 ± 0.22	34	1.30 ± 0.21	0.42
Striatal DaT binding	219	1.45 ± 0.35	78	1.25 ± 0.41	**0.0002**

Analysis was conducted to determine if participants lost to attrition, and therefore not included in cluster analysis, presented differences at baseline. In brief, participants lost to attrition performed worse in several domains, including significantly higher scores in MDS-UPDRS part 2, along with lower cognitive ability and serum IGF-1 concentrations (Table 3). While not significant following correction for multiple comparisons, participants without follow-up also trended towards having higher MDS-UPDRS part 1 and SCOPA-AUT scores and higher age, along with lower UPSIT scores.

**Table 3.** Baseline Characteristics of Participants Included in the Final Dataset, who all had MDS-UPDRS Scores up to Year-five Follow-up, Compared Against Participants Lost to Attrition. Overall, Participants Lost to Follow-up Demonstrated Significantly Worse Outcomes in Multiple Domains at Baseline. Normally Distributed Variables are Displayed as Mean ± SD, With Statistical Differences Determined via Independent Samples T-test. Non-Parametric Variables are Displayed as Median (IQR), With Differences Determined via Mann-Whitney U. Count Data are Presented as Percentages (%) and Differences were Assessed via a Chi-square Test. A Bonferroni Correction was Applied to Account for Multiple Comparisons, Setting the Threshold to P < 0.003. Statistically Significant Differences are Denoted by Bolded P-values.

	Participants with follow-up n = 300	Participants w/o follow-up n = 122	*P*-value
Age	61.82 (54.3 – 68.5)	64.05 (58.5 – 70.1)	0.013
Education	16 (14 – 18)	16 (14 – 17)	0.12
Sex			
Male	200 (66.7%)	77 (66%)	0.56
Female	100 (33.3%)	45 (34%)	
MDS-UPDRS part 1	5 (2 – 7)	6 (3 – 8)	0.023
MDS-UPDRS part 2	4 (2 – 8)	6 (4 – 9)	**0.002**
Rigidity score	3 (2 – 5)	3 (2 – 6)	0.99
Tremor score	4 (2 – 6)	4 (2 – 7)	0.18
Cognitive ability	0.17 (−0.47 – 0.80)	−0.19 (−0.97 – 0.37)	**<0.001**
Mood dysfunction	−0.28 (−0.73 – 0.44)	−0.26 (−0.65 – 0.45)	0.58
CSF α-syn	1374.1 (1034.4 – 1792.2)	1422.5 (1127 – 1758.3)	0.33
CSF p-tau	13.2 (11.13 – 17.12)	14.3 (11.6 – 18.3)	0.08
CSF Aβ	867.2 (642.1 – 1150.3)	831.7 (590.6 – 1053.3)	0.32
Serum IGF-1	135 (103.1 – 166.9)	109.8 (85.2 – 152.1)	**0.001**
UPSIT	22 (15.75 – 28)	23 (16 – 30)	0.30
RBDSQ	3 (2 – 5)	4 (2 – 6.5)	0.009
SCOPA-AUT	8 (5 – 12)	10 (6.3 – 13.8)	0.006
Striatal DaT binding	1.40 0.38	1.45 0.45	0.29

#### Year-Five Follow-up Characteristics

At year-five follow-up, cluster 2 consistently performed worse across all assessments, demonstrating a significantly higher MDS-UPDRS part 1, MDS-UPDRS part 2 and rigidity score compared to cluster 1, as well as higher prevalence of mood dysfunction. Additionally, cluster 2 demonstrated more profound postural instability, indicated by significantly higher Hoehn and Yahr scores, as well as a larger proportion of individuals with postural instability (score ≥3), albeit not significant following correction for multiple comparisons. Cluster 2 also demonstrated significantly lower cognitive ability, further corroborated by a much larger proportion of participants within cluster 2 reporting mild cognitive impairment (MCI) and dementia compared to cluster 1. Year-five follow-up comparisons between clusters are presented in Table 4.

**Table 4.** Differences Between Clusters in Assessments With follow-up Data Were Assessed at Year-five. Further, Clinical Characteristics, such as Hoehn Yahr Staging and Cognition-Related Diagnoses, were Compared. Non-parametric Variables are Displayed as Median (IQR), With statistical Differences Determined via Mann-Whitney U Tests. Count Data are Presented as Percentages (%) and Differences were Assessed via a Chi-square Test. A Bonferroni Correction was Applied to Account for Multiple Comparisons, Setting the Threshold to P < 0.006. Statistically Significant Differences are Denoted by Bolded P-values.

	Cluster 1 n = 221		Cluster 2 n = 79		*P*-value
	n	Mean ± SD/Median (IQR)	n	Mean ± SD/Median (IQR)	
MDS-UPDRS part 1	221	7 (4 – 10)	79	15 (11.5 – 20)	**<0.0001**
MDS-UPDRS part 2	221	7 (4 – 11)	79	17 (13 – 19)	**<0.0001**
Rigidity score	221	4 (2 – 6)	79	6 (3 – 8.5)	**<0.0001**
Tremor score	221	4 (0 – 7)	79	2 (0 – 3)	**<0.0001**
Cognitive ability	220	0.26 (−0.41 – 0.79)	79	−0.22 (−1.03 – 0.49)	**0.0003**
Mood dysfunction	221	−0.45 (−0.85 to −0.01)	79	0.41 (−0.17 – 1.50)	**<0.0001**
Hoehn and Yahr		2 (2 – 2)		2 (2 – 3)	**0.0008**
	≤2	211 (96%)	64 (81%)		0.007
	≥3	8 (4%)	15 (19%)		
Cognitive outcome					
Normal		150 (69%)		32 (41%)	**<0.0001**
Indeterminate		25 (11%)		12 (15%)	
Cognitive complaint		28 (13%)		18 (23%)	
MCI		11 (5%)		4 (5%)	
Dementia		4 (2%)		9 (11%)	

#### Rate of Progression

**Figure 3.**
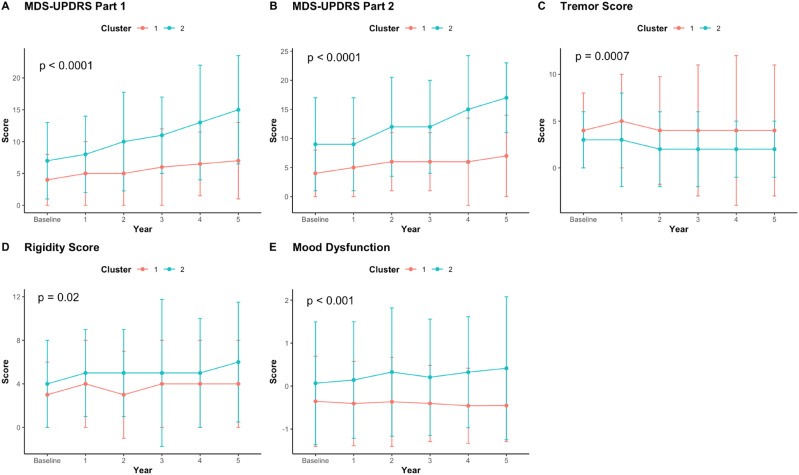
Median & IQR values at each timepoint displayed for variables with significant interaction effects in the non-parametric mixed ANOVA’s. Differences in slopes between clusters were assessed via Mann-Whitney U tests.

For variables with significant interaction effects (Table S4), slopes were calculated using all timepoints and compared across clusters via Mann-Whitney U tests (Figure 3). Cluster 2 demonstrated significantly higher rates of progression in various assessments, including MDS-UPDRS part 1 (*P* < 0.0001) and part 2 scores *(P <* 0.0001), as well as mood dysfunction (*P* < 0.0001). Further, cluster 2 demonstrated a significantly lower rate of progression in tremor scores (*P* = 0.0007). While cluster 2 displayed a trend towards more rapid progression in rigidity scores (*P* = 0.02), this did not reach significance after applying a correction for multiple comparisons (threshold: *P* < 0.01).

#### Cluster Membership Prediction

Logistic regression was conducted on participants with no missing data across all baseline predictor inputs (n = 111; cluster 1 = 85 & cluster 2 = 26) (Table 5). Demographic information on this subset is reported in Table S5.

**Table 5.** Two Logistic Regression Models Were Created With Cluster Membership as the Outcome Variable. The First Model Only Included Baseline Values of the MDS-UPDRS Scores (the Year-Five Values for the Same Measures Were Used in the Hierarchal Clustering Protocol) as Predictors, Whereas the Second Model Included Additional Baseline Demographic, Neuroimaging, Biofluid Marker and Prodromal Assessment Scores. Significant Predictors are Denoted by Bold *P-*Values, With Threshold Set to 0.05 as R Computes Internal Corrections for Multiple Comparisons.

Cluster membership						
*Predictors*	MDS-UPDRS assessment			Multi-modal assessment		
	*Odds Ratios*	*std. Beta*	*P*	*Odds Ratios*	*std. Beta*	*P*
Intercept	0.04	0.23	**<0.001**	0.38	0.10	0.849
MDS-UPDRS part 1 score	1.10	1.38	0.250	1.03	1.10	0.838
MDS-UPDRS part 2 score	1.32	2.76	**<0.001**	1.29	2.54	**0.018**
Rigidity score	1.03	1.07	0.799	0.93	0.83	0.680
Tremor score	0.96	0.89	0.661	0.99	0.97	0.940
Age				0.96	0.67	0.378
Female sex				0.18	0.43	0.072
Education (Years)				1.16	1.51	0.309
SN proxy marker				7.28	1.54	0.221
Striatal DaT binding				0.15	0.51	0.093
CSF α-syn^a^				0.68	0.09	**0.038**
CSF p-tau				1.49	6.45	**0.023**
CSF Aβ^a^				1.05	1.16	0.741
Serum IGF-1				1.00	1.07	0.845
RBDSQ				1.10	1.26	0.501
UPSIT				0.92	0.47	**0.040**
SCOPA-AUT				1.25	3.64	**0.026**
Observations	111			111		
R^2^ Tjur	0.274			0.491		

^a^CSF α-syn and Aβ were rescaled to improve interpretability, with odds ratios reflecting effects per 100 pg/mL. CSF p-tau was not rescaled, as its unit was already interpretable.

**Figure 4.**
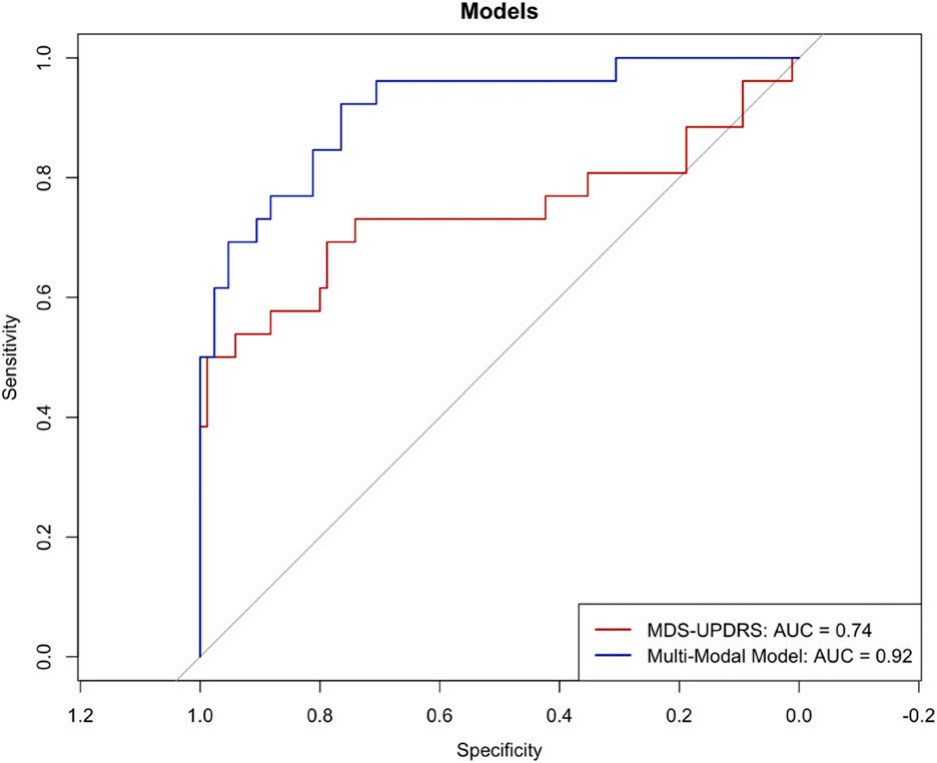
ROC curves were created for each model to compare performance. The multi-modal model (blue line) resulted in an AUC of 0.92 (sensitivity = 0.92, specificity = 0.75), while the model using only baseline MDS-UPDRS assessments (red line) achieved an AUC of 0.74 (sensitivity = 0.5, specificity = 0.99). The diagonal dotted line represents chance-level performance (AUC = 0.50).

When only including baseline MDS-UPDRS assessments, the only significant predictor of cluster membership was MDS-UPDRS part 2 score, with an overall predictive value of 27.4% (R^2^ = 0.274). Similarly, MDS-UPDRS part 2 score remained a significant predictor in the multi-modal assessment model, along with CSF levels of both *α*-syn and p-tau, UPSIT score and SCOPA-AUT score, with the latter being reflected by significant differences in baseline scores between clusters. The additional predictors included in model 2 increased the proportion of variance explained by 21.7% relative to model 1, and this difference was significant (F (12, 94) = 29.25, *P* = 0.004). Additionally, ROC curve analyses corroborated such findings, with the multi-modal model corresponding to an AUC of 0.92 (sensitivity = 0.92, specificity = 0.75), while the model only including baseline MDS-UPDRS assessments resulted in an AUC of 0.74 (sensitivity = 0.5, specificity = 0.99), as shown in Figure 4. Relative importance of predictors for both models are presented in Figures S6 & S7.

#### Machine Learning

Validation scores determined that the best classifiers for MDS-UPDRS assessment, in isolation and multi-modal assessment, were SVM and RFC respectively (Table 6).

**Table 6.** Balanced Accuracy Scores Calculated Using 5-Fold Cross-Validation Were Used to Quantify the Results of Each Classifier per Input Set. The Classifier With the Highest Validation Score in Each Case, as Denoted by Bold Values, was Selected as the Best Classifier for Model Development.

	SVM	KNN	RFC
MDS-UPDRS assessment	**0.75**	0.67	0.70
Multi-modal assessment	0.66	0.57	**0.72**

Balanced accuracy scores were obtained from the selected classifier in each case, for each dataset partition (training, validation, test). In support of both the logistic regression and ROC findings, while both assessments showed higher classification accuracy compared to chance performance, the multi-modal assessment (validation score = 0.67 ± 0.04) had a higher test result of 0.74, compared to a score of 0.60 for the assessment using MDS-UPDRS alone (validation score = 0.75 ± 0.05).

## Discussion

Currently, PD diagnosis and prognosis are largely reliant on clinical criteria, such as the MDS-UPDRS, corresponding to several shortcomings, such as high rates of misdiagnosis. A possible strategy to mitigate this and advance understanding of PD outcomes is to utilise a multi-modal combination of markers, incorporating not only the MDS-UPDRS, but also biofluid and neuroimaging-based biomarkers, as well as more comprehensive clinical evaluations, such as prodromal assessments. In this study, the combination of such markers resulted in better prediction of membership in the more impaired cluster at year-five follow-up, and improved classification accuracy in utilised machine learning techniques.

Hierarchal clustering on MDS-UPDRS scores at year-five follow-up identified 2 clusters. Retrospective assessment determined cluster 2 demonstrated significantly higher rigidity at year-five follow-up, as well as a faster rate of progression in rigidity over time (albeit not statistically significant after applying multiple comparison correction), along with a significantly slower progression in tremor scores. Additional features reported across the motor subtyping literature were also identified, including older age of onset,^45,46^ albeit not statistically significant following multiple comparison correction, and poorer functional outcomes, measured by MDS-UPDRS Part 1 and 2 scores,^47^ in the more-rigid cluster at both baseline and year-five follow-up. Additionally, cluster 2 demonstrated significantly higher scores on the Hoehn and Yahr scale. Overall, this suggests the clusters identified are similar to clinically recognised subtypes of PD, with cluster 1 reflecting tremor dominant (TD), whereas cluster 2, displaying the aforementioned features, is more similar to clinically relevant motor phenotypes, namely akinetic-rigid- (AR) or PIGD-PD.^48^

However, it should be noted that there was no significant difference in either tremor and rigidity scores between clusters at baseline, and neither measure acted as a significant predictor in the logistic regression models predicting cluster membership. In fact, of the baseline MDS-UPDRS assessments, only part 2 total score significantly predicted cluster membership, both in the model with MDS-UPDRS scores used in isolation as predictors and in the multi-modal model. Additionally, MDS-UPDRS measurements, with the exception of part 2 total score, presented with relatively weak prognostic utility, further supported by the findings of the relative importance plots. Interestingly, this was despite the potential risk of circularity of the present approach, where clusters were derived via year-five MDS-UPDRS scores and cluster membership was assessed retrospectively against the baseline predictors. This further corroborates previous reports in the literature that MDS-UPDRS derived scores at time of diagnosis are not reflective of future assessment outcomes,^5^ and highlights the need to extend prediction of disease progression beyond the MDS-UPDRS alone.

Differences in the progression of cognition and mood were also explored between clusters. Consistent with the literature, the more-rigid cluster had significantly lower cognitive ability scores, both at baseline and year-five follow-up.^49^ The more-rigid cluster also demonstrated higher rates of both dementia diagnosis and subjective cognitive complaints at year-five follow-up compared to the more TD-dominant cluster (although not higher rates of MCI diagnosis). Disparate risk for dementia conversion between motor subtypes is widely recognised in the PD literature,^50^ with a possible explanation being distinct pathophysiologies. Interestingly, however, there was no difference in rate of cognitive decline between the 2 clusters. This could be attributed to difficulties in assessing cognition, particularly in early PD. This is highlighted in a meta-analysis exploring PD conversion into PD-mild cognitively impaired and PD-dementia, where high reversion rates were seen within the first 3 years of diagnosis.^51^ Another important consideration is that the current clustering technique utilised total MDS-UPDRS part 1 scores, rather than addressing specific sub-domains that comprise non-motor function. For example, severity of psychosis is a recognised predictor of multidomain cognitive decline,^52^ but was likely not captured with the current cluster definitions. Therefore, future studies should explore specific non-motor domains as predictors of cognitive change in PD.

In contrast to our cognitive findings, the more-rigid cluster demonstrated significantly higher mood dysfunction both at baseline and year-five follow-up, as well as a faster rate of progression over the 5-year period following diagnosis. This aligns well with previous literature, where mood dysfunction, such as depression and apathy, is more profound in non-TD subtypes.^53^

Interestingly, despite significant differences in baseline clinical assessments, no baseline differences in biomarker concentrations were found between clusters. This is inconsistent with previous literature, in which different subtypes have been shown to display distinct biomarker profiles. For example, compared to TD-subtypes, plasma levels of α-syn and Aβ have been shown to be significantly higher and lower, respectively,^54^ with CSF concentrations of CSF p-tau shown to be higher,^55^ in PIGD-PD. This inconsistency may be because the PPMI dataset at baseline is highly homogenous.^56^ Although previous work in this dataset has shown baseline differences in biomarkers, such as CSF α-syn, between individuals with PD and healthy controls,^57^ it may be more difficult to detect differences between subtypes. Alternatively, this may be due to the restricted sample size of the current cohort, as only cases that had data for all variables of interest could be included in the analysis.

Nevertheless, a lack of significant differences in these biomarkers at baseline does not necessarily equate to poor prognostic utility. In support of this, lower levels of CSF α-syn and higher levels of CSF p-tau (but not CSF levels of Aβ or serum levels of IGF-1) were significant predictors of cluster membership within the multi-modal logistic regression model, both indicative of more advanced pathology.^58,59^ This is similar to previous literature, where reductions in CSF α-syn over time were associated with worsening motor function, particularly in those of the AR-subtype, in the DATATOP cohort^60^ (although see^61-63^). Similarly, CSF α-syn also displayed predictive value for change in cognitive function (although, interestingly, not of motor symptoms) in another study analysing data from the DATATOP cohort.^64^ Of note, higher CSF α-syn was associated with a faster rate of cognitive decline, contrary to what might be expected and inconsistent with results of the current work, where no difference in progression of cognitive symptoms was noted between clusters.

Regarding p-tau, emerging evidence suggests that p-tau plays a critical role in PD pathophysiology and may interact with α-syn to influence its pathology (for review, see^65^). Indeed, a recent study by Chu and colleagues^66^ (2024) demonstrated that tau, rather than α-syn aggregation, may mediate nigrostriatal DA neuron degeneration. Despite this, few studies to date have examined the relationship between PD progression and p-tau. One study showed no association between CSF p-tau levels and cognitive decline over time in a prospective cohort study (n = 45) with at least 1 yearly follow-up.^67^ More recent work, however, assessing plasma extracellular vesicles from 103 individuals with PD showed that elevated baseline plasma extracellular vesicle tau levels corresponded to significantly greater decline in both motor function and cognition.^68^

It should also be acknowledged that such biomarkers may have enhanced predictive utility when considered together. In light of this, within the PPMI cohort, a recent study examined whether the amyloid/tau/neurodegeneration (ATN) framework modified for PD (ATN_PD_), consisting of CSF Aβ (A) and p-tau_181_ (T) and serum neurofilament light (N), could predict longitudinal cognitive decline in 364 patients with PD and 168 age- and sex-matched controls.^69^ The study found that those classified as A + T+N+ had greater decline than all other ATN_PD_ groups over a 5-year follow-up period.^69^ This is also in line with a recent study from our group within PPMI, which found that decreased levels of CSF Aβ and increased CSF concentration of p-tau at baseline improved prediction of membership in a cluster with more impaired cognitive and affective function at 5-year follow-up, relative to inclusion of baseline cognitive and mood measures alone.^34^ This suggests further work in this area is warranted, in order to investigate how specific patterns of biomarker expression may drive distinct disease progression patterns.

Though biological fluid-based biomarkers demonstrated prognostic potential, the use of selected neuroimaging measures was less promising, which is in contrast to previous literature establishing links between such markers and PD progression. While the more-rigid cluster displayed significantly lower striatal DaT binding at baseline compared to the tremor cluster, no significant difference in the SN-related hypointensity measure between clusters was noted. Further, neither neuroimaging modality were significant predictors of cluster membership in the multi-modal model. Despite studies reporting significant correlations between baseline DaT and disease staging^70^ and rigidity severity,^71^ our findings reflect those reported by Chahine et al,^72^ whereby model performance to predict MDS-UPDRS change was noticeably low with baseline measures of DaT imaging. This was ameliorated by incorporating a short-term change to DaT binding as a predictor, a potential avenue moving forward. Regarding the MRI marker, a potential issue with the delineation protocol is assessing ROIs in 2D rather than 3D, leading to a potentially inaccurate representation of structural changes. Additionally, T2-weighted MRI may lack the sensitivity required to distinguish SN-related differences between clusters (see review,^73^). In support of this, the SN-related hypointensity did not correlate with motor outcomes or disease severity in a previous study.^74^ Instead, alternative imaging sequences may prove more beneficial for capturing differences between PD phenotypes. For example, AR-PD demonstrates more distinct pathological features in the SN compared to TD-PD, including increased regional iron deposition, as measured by quantitative susceptibility mapping,^75^ and more profound decline of neuromelanin, as measured by T1 neuromelanin-sensitive MRI.^76^ Thus, to probe further whether changes in neuroimaging markers are predictive of disease progression in PD, future studies should employ sequences sensitive to such pathological changes in PD, which were not available in the current work.

Outside of pathophysiological assessments, clinical assessments encompassing a wide array of prodromal symptoms show high promise for predicting risk of PD development,^77^ and therefore may show promise for prognosis of clinical progression. Here, the results of the multi-modal logistic regression highlight worse UPSIT and SCOPA-AUT scores significantly predicted membership in the more-rigid cluster, reflected by significant differences in such scores between clusters at baseline. Supporting this, He et al reported patients presenting hyposmia display a worse clinical course^78^ and are more likely to be classified as PIGD-dominant.^78,79^ Similarly, autonomic dysfunction, as measured by SCOPA-AUT, has consistently been reported to be higher in PIGD-PD compared to TD-PD^80,81^ and even intermediate subtypes.^81^ However, interestingly, the RBDSQ was not a significant predictor in this model, consistent with other studies reporting a lack of association between RBD or sleep disturbance and PIGD.^82,83^ Despite promise, it should be noted that prodromal symptoms are still largely underutilised clinically, due to non-specificity (see review^84^). Therefore, their utility may be highest when used in conjunction with more validated assessments, as seen in this study.

Overall, the findings of this study suggest that incorporating a multi-modal panel including biological measures, prodromal assessments, and current clinical criteria displays high prognostic utility for disease progression, beyond MDS-UPDRS in isolation. In regression models, baseline MDS-UPDRS assessment alone could only account for 27.4% of variance (AUC = 0.74), whereas the incorporation of neuroimaging, biofluid pathological markers, prodromal symptom presentation and key demographic variables increased the proportion of the variance explained to 49.1% (AUC = 0.92). This was corroborated by machine learning, where MDS-UPDRS assessment alone had a classification accuracy close to chance (60%), increasing to 74% when utilising multi-modal assessment. Further, while the multimodal assessment demonstrated lower validation scores, its higher test result suggests it performs more robustly on unseen data, indicative of higher generalisation ability, which is crucial for clinical translatability. However, for the machine learning, the test and validation scores were substantially different and inconsistent, suggesting a considerable amount of variance, requiring future testing in new data to assess this.

While promising, several limitations must be considered when interpreting findings from current work. Firstly, while data-driven clustering shows high promise, a systematic review has highlighted the difficulty in comparing across studies and raised concerns that true replications of clinical phenotypes are not reproducible.^28^ Therefore, harmonisation of clustering protocols is essential to make effective comparisons. Secondly, lack of available follow-up data for variables, such as biomarkers and prodromal features, made their incorporation into the data-driven subtyping unfeasible, as it would have greatly truncated sample size. Finally, between baseline and year-five, approximately 30% of participants were lost to follow-up and therefore excluded. Interestingly, compared to those included in the current analysis, participants lost to follow-up demonstrated considerably worse outcomes in various domains at baseline, including MDS-UPDRS scores, cognitive ability and prodromal assessments (eg, SCOPA-AUT). As a result, the progression severity of cluster 2 is likely to be underestimated, which may introduce attrition bias. Specifically, the effects of prognostic utility of baseline variables reported within this study may underestimate those seen in the wider PPMI dataset.

Additionally, while the PPMI dataset is a rich and comprehensive resource, its cohort present with certain demographics that may make them less generalisable to the wider population. Marek and colleagues reported that individuals included in the PPMI dataset are generally highly educated, as well as predominantly Caucasian.^85^ In fact, the limitation of participants being younger, better educated and healthier than the broader population is not just a limitation of PPMI, but also of similar large datasets, namely DATATOP.^86^ To ensure analyses conducted within the PPMI dataset are translatable, external replication is required^87^; however, to the best of our knowledge, no other publicly available dataset currently offers the scope of measures available in PPMI, particularly with annual longitudinal follow-up over an extended period. We attempted to mitigate this limitation by performing stratified 5-fold cross-validation within the utilised dataset, demonstrating predictive performance above chance on a test set not included as part of model training. While this strengthens confidence in the results of the current study, it is nevertheless critical that future work seek to replicate these findings in independent cohorts where possible.

## Conclusion

Current diagnostic criteria for PD rely heavily on clinical judgment, with misdiagnosis occurring in a significant percentage of individuals. Further, the use of the MDS-UPDRS alone for long-term prognosis in early PD cohorts has limitations, perhaps because the MDS-UPDRS is not comprehensive enough to capture the heterogeneity of PD progression. In the current study, incorporating additional assessments, primarily pathological biomarkers, substantially improved sub-type classification and may provide key insight into differential outcomes across PD. Continued exploration into underlying pathophysiology of PD will advance development of prognostic models, which could greatly enhance more personalised management of PD.

## Supplemental Material

Supplemental Material - Utility of Baseline Pathological, Neuroimaging and Clinical Markers for Prognosis in Early Parkinson’s Disease Supplemental Material for Utility of Baseline Pathological, Neuroimaging and Clinical Markers for Prognosis in Early Parkinson’s Disease by Angus McNamara, Benjamin Paul Ellul1 ORCID, Irina Baetu, Mark Jenkinson, Stephan Lau and Lyndsey Collins-Praino in Journal of Geriatric Psychiatry and Neurology

## Consent to Participate

Written informed consent were obtained from each participant at enrolment, in accordance with the Declaration of Helsinki. All methods were performed in accordance with the relevant guidelines and regulations. We confirm that we have read the Journal’s position on issues involved in ethical publication and affirm that this work is consistent with those guidelines.

## Data Availability

All data analysed within the current study are available within the PPMI database, https://www.ppmi-info.org/access-data-specimens/download-data.
