# Supplementary material for: Utility of Baseline Pathological, Neuroimaging and Clinical Markers for Prognosis in Early Parkinson’s Disease
Source: J Geriatr Psychiatry Neurol. 2025 Nov 13;39(4):471–87. doi: 10.1177/08919887251397641 (PMC13103341; doi:10.1177/08919887251397641)
Supplement: Supplemental Material - Utility of Baseline Pathological, Neuroimaging and Clinical Markers for Prognosis in Early Parkinson’s Disease [file sj-pdf-1-jgp-10.1177_08919887251397641.pdf]

Table S1: *Inclusion and exclusion criteria that PPMI have developed to categorise participants into the early PD cohort.*

**Inclusion Criteria**	**Exclusion Criteria**
1) Presence of 2 or more cardinal motor symptoms:<br>1. Resting tremor<br>2. Bradykinesia<br>3. Rigidity<br>OR<br>a) Asymmetric resting tremor<br>OR<br>b) Asymmetric bradykinesia<br>&<br>2) Dopamine transporter deficit on SPECT imaging<br>&<br>3) Diagnosis of PD < 2 years before baseline visit	First-degree relative of a PD patient (Genetic cohort)<br><br>Patients with scans without evidence of dopaminergic deficit (SWEDD cohort)<br><br>< 30 years old

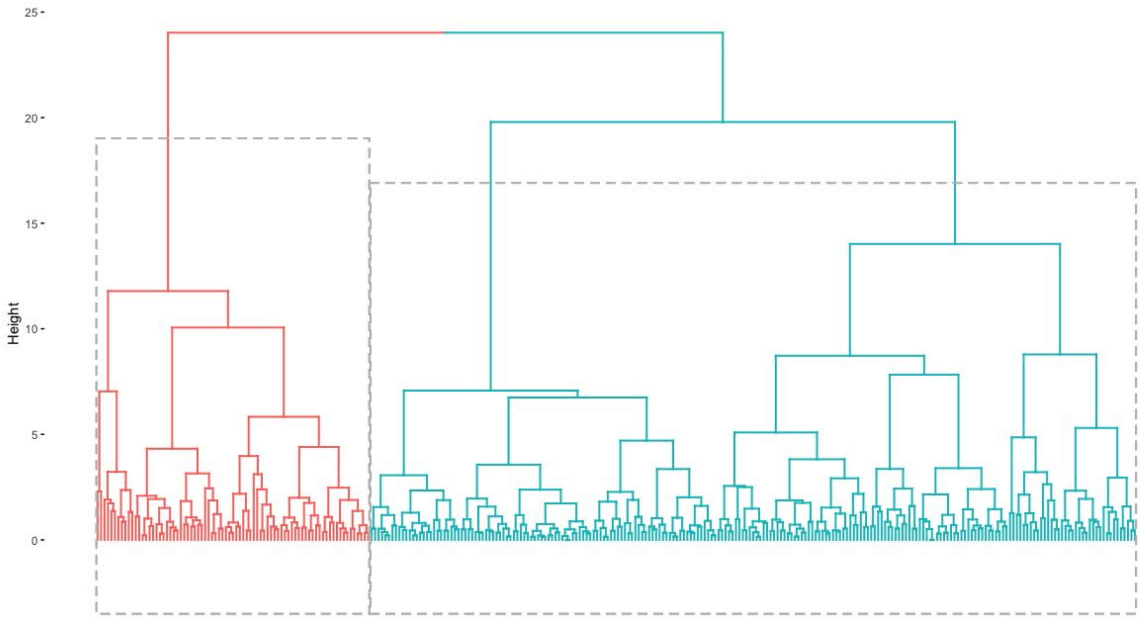

Figure S2: *Dendrogram detailing cluster delineation using UPDRS assessments at year-5 follow-up.*

Table S3: *PCA was conducted on cognitive assessments and mood-related assessments to provide composite scores based on the first unrotated factor. This was conducted on baseline values as well as on beta coefficients that estimated the slope of change in mood or cognition over the follow-up period.*

	**Loadings**	**Eigenvalue**	**Variance Accounted by Factor**
**Baseline Cognitive Ability PCA**		2.27	45%
HVLT	.69		
LNS	.72		
SFT	.71		
BJLOT	.48		
SDM	.74		
**Baseline Mood Dysfunction PCA**		1.65	83%
STAI	.91		
GDS	.91		
**Cognitive Ability Beta Coefficient PCA**		2.17	77%
HVLT slope	.74		
LNS slope	.66		
SFT slope	.61		
BJLOT slope	.58		
SDM slope	.69		
**Mood Dysfunction Beta Coefficient PCA**		1.62	91%
STAI slope	.9		
GDS slope	.9		

Table S4: *Non-parametric equivalent of Mixed ANOVAs with a between-subjects factor (cluster membership) and a within-subjects factor (year) were conducted. Of primary interest was whether significant interaction effects were present to determine whether clusters differed in rates of progression*

	**Cluster**	**Year**	**Interaction Effect**
**MDS-UPDRS Part 1**	F(1, 298) = 138.18<br>**P < 0.001**	F(1, 298) = 254.72<br>**P < 0.001**	F(1, 298) = 21.45<br>**P < 0.001**
**MDS-UPDRS Part 2**	F(1, 298) = 193.46<br>**P < 0.001**	F(1, 298) = 216.28<br>**P < 0.001**	F(1, 298) = 11.61<br>**P < 0.001**
**Rigidity Score**	F(1, 298) = 22.42<br>**P < 0.001**	F(1, 298) = 22.77<br>**P < 0.001**	F(1, 298) = 5.58<br>**P = 0.02**
**Tremor Score**	F(1, 298) = 21.13<br>**P < 0.001**	F(1, 298) = 23.69<br>**P < 0.001**	F(1, 298) = 12.10<br>**P < 0.001**
**Cognitive Ability**	F(1, 292) = 12.23<br>**P < 0.001**	F(1, 292) = 2.71<br>P = 0.1	F(1, 292) = 0.34<br>P = 0.56
**Mood Dysfunction**	F(1, 296) = 40.01<br>**P < 0.001**	F(1, 296) = 4.85<br>**P = 0.02**	F(1, 296) = 24.05<br>**P < 0.001**

Table S5: *Demographic information for the subset of participants part of the final logistic regression models, based on data completeness. Data are displayed as median (IQR), with differences determined via Mann-Whitney U. Qualitative data were presented as counts (%) and differences were assessed via a Chi-square test. Statistically significant differences are denoted by bolded P-values.*

	**Cluster 1** n = 85	**Cluster 2** n = 26	**P-Value**
**Age**	61.16 (53.8 – 67.7)	63.51 (55.86 – 70.9)	0.21
**Education**	16.00 (14 – 18)	16.00 (14 – 18)	0.99
**Sex**			
Females	36 (42)	5 (19)	0.06
Males	49 (58)	21 (81)	

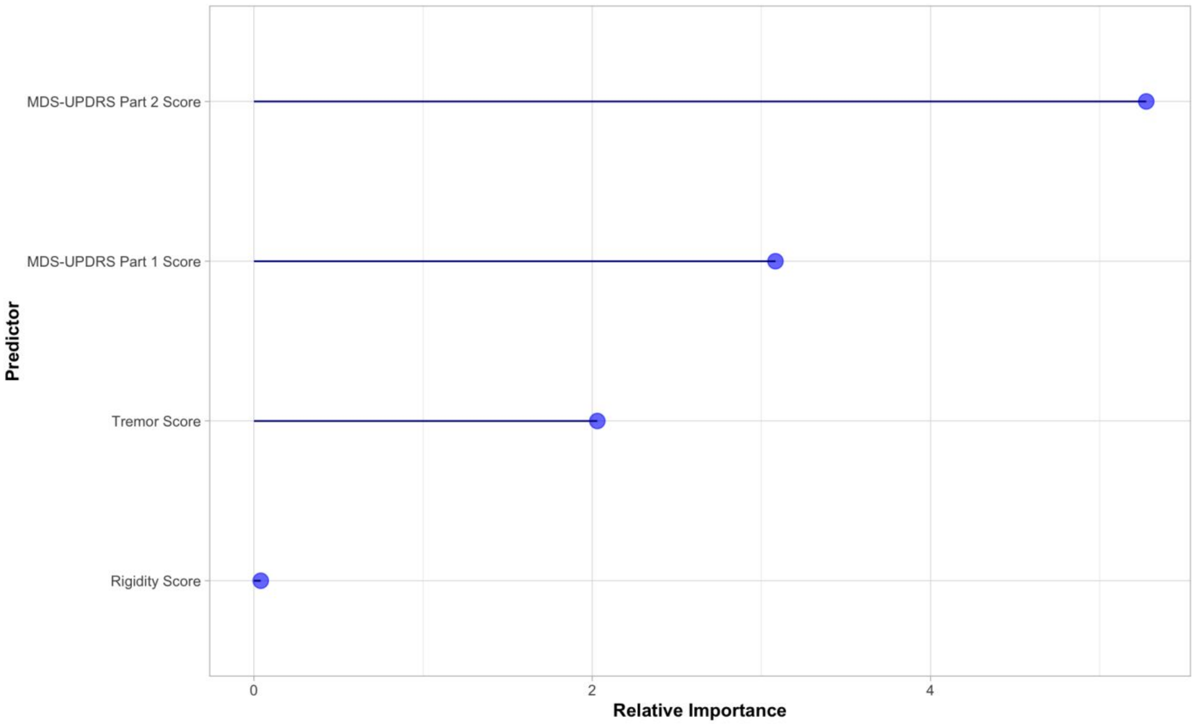

Figure S6: *Relative importance of predictors in the logistic regression model exploring cluster membership as the outcome, which only included baseline UPDRS assessments. Values on the x-axis should be interpreted as a representation of the overall variance explained by predictors in reference to one another.*

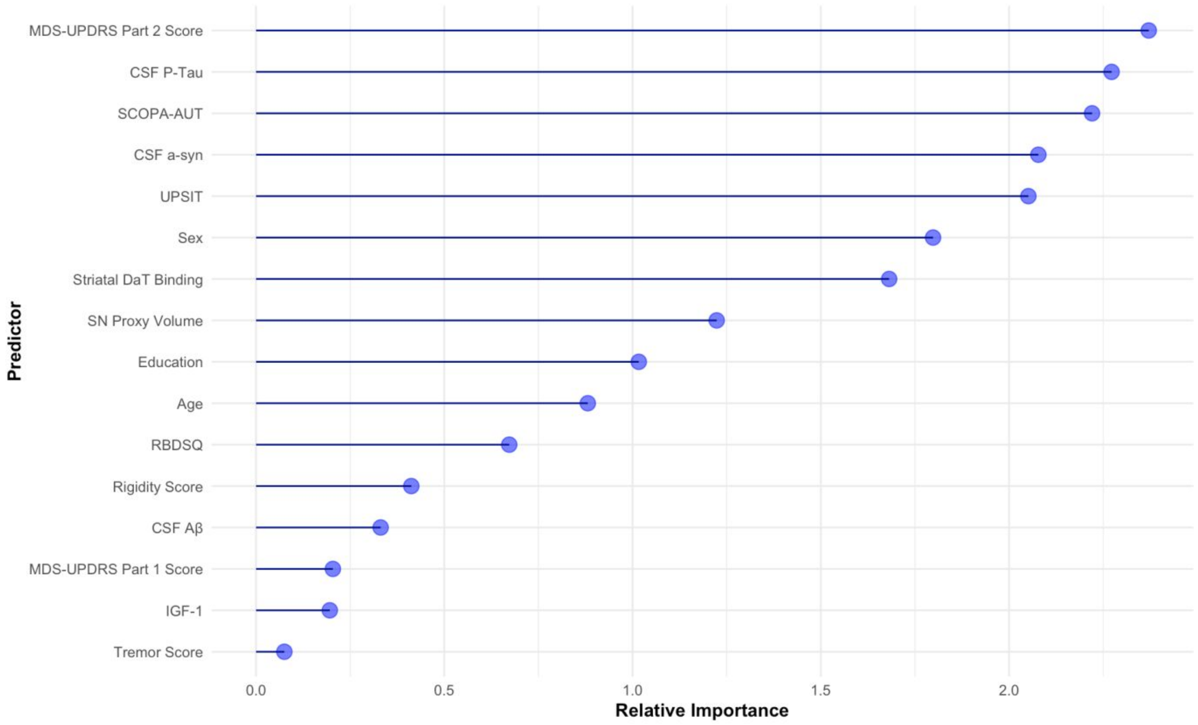

Figure S7: *Relative importance of predictors in the multi-modal logistic regression model exploring cluster membership as the outcome, including baseline UPDRS scores and additional predictors, including various biomarkers, neuroimaging modalities and clinical assessments of prodromal symptoms. Values on the x-axis should be interpreted as a representation of the overall variance explained by predictors in reference to one another.*
